# YTHDF2 Regulates Maternal Transcriptome Degradation and Embryo Development in Goat

**DOI:** 10.3389/fcell.2020.580367

**Published:** 2020-09-29

**Authors:** Mingtian Deng, BaoBao Chen, Zifei Liu, Yu Cai, Yongjie Wan, Guomin Zhang, Yixuan Fan, Yanli Zhang, Feng Wang

**Affiliations:** ^1^Jiangsu Livestock Embryo Engineering Laboratory, College of Animal Science and Technology, Nanjing Agricultural University, Nanjing, China; ^2^College of Animal Science and Technology, Nanjing Agricultural University, Nanjing, China; ^3^College of Veterinary Medicine, Nanjing Agricultural University, Nanjing, China

**Keywords:** embryogenesis, maternal-to-zygotic transition, N6-methyladenosine, YTHDF2, maternal mRNA decay, zygotic genome activation

## Abstract

Maternal mRNA clearance is critical for the early embryo development, which is under the tight control of RNA N6-methyladenosine (m^6^A). However, little information is known regarding the maternal mRNA clearance and mechanisms behind it in farm animals. In the present study, 3362 differentially expressed genes (DEGs) were found during the maternal-to-zygotic transition (MZT) and determined as maternal mRNAs in goat. Of which, 1961 was decreased at the 4-cell stage embryos, while 1401 was trigged down-regulation at the 8-cell stage embryos, which were termed as maternally encoded mRNA decay genes and zygotic genome activation (ZGA)-dependent maternal mRNAs, respectively. The expression of m^6^A reader YTHDF2 was increased during goat ZGA, and knockdown of YTHDF2 resulted in decreased blastocyst rate. In the 8-cell stage YTHDF2 knockdown embryos, the M-decay and Z-decay maternal mRNA clearance were impaired. Specifically, the expression of deadenylase (*CNOT1* and *CNOT11*) and decapping enzymes (*DCP1A* and *DCP2*) was decreased. In conclusion, we ascertained maternal mRNAs and inferred that maternal mRNA clearance is also ZGA-dependent in goat. We reported that YTHDF2 is vital for goat early embryogenesis as it advances maternal mRNA clearance, which might through the recruitment of deadenylases and mRNA decapping enzymes. This work will be of great value for understanding the stochastic reprogramming events during MZT and achieving better development of goat embryos *in vitro*.

## Introduction

The early stages of embryogenesis in mammals takes place from the fertilization, during which, the majority of maternal mRNAs undergo rapid degradation and developmental control is handed from maternally provided gene products to those synthesized from the zygotic genome through a process known as maternal-to-zygotic transition (MZT). During MZT, most of the maternal mRNAs were eliminated, but ∼10% genes of the genome sustain expression, which are also essential for early embryo development. For example, knockout of methyltransferase like 3 (METTL3), lysine-specific demethylase 5B (KDM5B), and lysine-specific demethylase 1A (LSD1) causes developmental arrest at the zygotic genome activation (ZGA) stage in mice (Ancelin et al., 2016; Dahl et al., 2016) and zebrafish (Xia et al., 2018). These and subsequent studies demonstrated that maternal gene products are essential for embryogenesis (Ancelin et al., 2016; Dahl et al., 2016; Xia et al., 2018; McDaniel et al., 2019).

One of the other processes of MZT is ZGA. It has been well documented that ZGA mainly begins at the 2-cell stage in mice, and at the 4- to 8-cell stage in human, pig, bovine, and goat (Tadros and Lipshitz, 2009; Palfy et al., 2017; Deng et al., 2018). Developmental arrest was often observed during ZGA in embryos cultured *in vitro*, and increasing studies revealed that block of ZGA causes improper gene expression and embryonic lethality (Lee et al., 2014; Abe et al., 2018), suggesting that the onset of ZGA is pivotal for the early embryo development.

RNA N6-methyladenosine (m^6^A) is critical for translation efficiency, alternative splicing, and mRNA stability (Yue et al., 2015). The m^6^A is instructed through recruit or displace defined RNA-binding proteins, such as IGF2BP proteins, the translation initiation factor eIF3 complex, and the HNRNP family members (Yue et al., 2015). In addition, m^6^A functions by direct recognition of YT521-B Homology (YTH) domain-containing proteins (Liao et al., 2018). Five YTH domain-containing proteins, YTHDC1, 2 and YTHDF1, 2, 3, were reported. YTHDC1 localizes to the nucleus, while YTHDC2, YTHDF1, YTHDF2, and YTHDF3 are expressed in the cytoplasm (Zhang et al., 2020). It was reported that YTHDC1 deficiency causes massive alternative splicing defects in mouse oocytes (Kasowitz et al., 2018), whereas YTHDC2 is required for spermatogenesis and oogenesis in mice (Hsu et al., 2017). Specifically, YTHDF2 is essential for the early zebrafish embryonic development and mouse oocyte competence (Ivanova et al., 2017; Zhao et al., 2017). Therefore, m^6^A plays important roles during early embryo development.

Recognition of m^6^A mRNA by YTHDF2 within the GACU/A consensus is associated with mRNA destabilization and degradation (Du et al., 2016), suggesting that maternal mRNA clearance is also under the tight control of m^6^A. Indeed, an increasing number of studies demonstrated that knockout of IGF2BP2 (Liu et al., 2019), YTHDC2 (Hsu et al., 2017), and YTHDF2 (Zhao et al., 2017) impaired maternal mRNA elimination. Moreover, zygotic transcription is first detected at the late 1-cell stage, whereas the majority of maternal mRNAs are removed by the 2-cell stage in mice (Sha et al., 2019a; Vastenhouw et al., 2019), suggesting that there is a complex crosstalk of maternal and zygotic products in regulating MZT, thus ensuring timely transfer of developmental control (Sha et al., 2019b).

Goat has been specifically an interesting subject due to its high value in biopharmaceutical and agricultural economy. However, goat embryo development is low and little information is known regarding the maternal mRNA clearance and mechanisms behind it during embryogenesis. In the present study, we determined 3362 differentially expressed genes (DEGs) as goat maternal mRNAs using RNA-seq. We further reported that knockdown of *YTHDF2* led to decreased blastocyst rate and impaired the maternal mRNAs clearance by downregulation of decapping enzymes *DCP1A* and *DCP2*, and the deadenylase *CNOT1* and *CNOT11*.

## Materials and Methods

### *In vitro* Maturation

Ovaries were collected from a local slaughterhouse, and *in vitro* maturation (IVM) was performed as previously described (Deng et al., 2019). Briefly, ovarian follicles 2–6 mm in diameter were punctured using a blade. The follicular fluid was flushed into a 60-mm petri dish using DPBS plus 1% fetal bovine serum (FBS). Then cumulus-oocyte complexes (COCs) with more than two layers of compact cumulus cells and a dense, homogeneous cytoplasm were rapidly selected under a stereomicroscope and washed five times with IVM medium (TCM-199 supplemented with 10% FBS, 1% FSH, 1% LH and 0.2% oestradiol). Subsequently, 15 of the COCs were cultured in a 60-μL droplet of IVM medium at 38.5°C, 5% CO_2_, 95% air, and saturated humidity for 20–22 h. Matured oocytes were then repeatedly pipetted with 0.3% hyaluronidase to remove the surrounding cumulus cells, and those with clear perivitelline spaces, intact cell membranes and extruded first polar bodies were selected for *in vitro* fertilization (IVF).

### *In vitro* Fertilization

*In vitro* fertilization was performed as previously described (Deng et al., 2020). Briefly, 20 of IVM COCs were transferred to a 75-μL droplet of BO-IVF medium (IVF Bioscience, Falmouth, United Kingdom) with mineral oil covering the surface, and freshly collected sperm was diluted and suspended to 2 to 9 × 10^6^ spermatozoa/mL. Then, 50 μL of the sperm suspension was added to the droplets and further cultured for 16 h at 38.5°C with 5% CO_2_, 5% O_2_, 90% N_2_, and saturated humidity. The remaining spermatozoa and cumulus cells were dispersed by gentle blowing with a pipette, and zygotes were transferred into a 75-μL droplet of BO-IVC medium (IVF Bioscience) supplemented with 10% FBS.

### Embryo Culture

Embryos were washed three times with BO-IVC medium supplemented with 10% FBS and cultured in groups of 20–25 in 75-μL droplet of BO-IVC medium supplemented with 10% FBS at 38.5°C with 5% CO_2_, 5% O_2_, 90% N_2_, and 100% humidity. They were collected at 72 h post-IVF to obtain embryos for gene expression analysis and RNA-seq.

### Knockdown of *YTHDF2*

Small interfering RNAs (siRNAs) against *YTHDF2* were formulated using the BLOCK-iT RNAi designer tool (refer to Supplementary Table S1) and synthesized at GenePharma (Shanghai, China). In general, 5–10 pL of 20 μM siRNAs targeted *YTHDF2* were microinjected into the cytoplasm of zygotes at 8 h after IVF. The MISSION siRNA universal negative control was served as a negative control (NC) for knockdown experiments. These microinjected zygotes were cultured in BO-IVC medium supplemented with 10% FBS at 38.5°C, 5% CO_2_, 5% O_2_, 90% N_2_, and 100% humidity. Development status was determined at 72 and 168 h after microinjection.

### Gene Expression Analysis

Complementary DNA (cDNA) from pooled embryos (*n* = 5) was synthesized using cellAmp whole transcriptome amplification kit (Takara, Dalian, China) following the manufacturer’s instruction. Briefly, embryos were directly lysed and used as a temple to synthesize the first-strand of cDNA, and poly(dA) tail was added using TdT enzyme mix and dATP at 37°C for 15 min. Afterward, second-strand cDNA was synthesized using dNTP mix and Ex Taq Hot Start, and the cDNA was amplified using Ex Taq Hot Start. Quantitative PCR (qPCR) was performed on an ABI 7300 Real-Time PCR System. All qPCR reactions were performed in triplicate, as described previously (Deng et al., 2018). Relative mRNA expression was normalized to *Gapdh* and calculated using the 2^–Δ^ ^Δ^ ^*Ct*^ method. Primers used in qPCR are shown in Supplementary Table S2. Moreover, the RNA-seq data of bovine and human embryos were downloaded from GEO: GSE59186 and GSE36552, respectively. The expression of *YTHDF2* in bovine and human was analyzed.

### RNA Library Construction and Sequencing

Embryos were directly lysed and used for cDNA synthesis using the Smart-seq2 method (Picelli et al., 2014). To profile RNA expression in 8-cell stage *YTHDF2* knockdown embryos and controls, 15 of each were pooled and directly lysed. Two replications were performed. cDNA was fragmented by dsDNA fragmentase (NEB, M0348S) by incubating at 37°C for 30 min, and size selection was performed with provided sample purification beads, then the fragmented cDNA at the size of 150–300 bp was used for library construction. And then, the paired-end sequencing was performed on an Illumina NovaSeq 6000 platform (LC Sciences, United States) following the vendor’s recommended protocol.

### Read Alignment and Differential Expression Analysis

RNA-seq data of MII oocytes and 4- and 8-cell staged goat embryos were downloaded from our previous studies (Deng et al., 2018; Liu et al., 2020). The generated reads were aligned to goat genome ARS1 using HISAT2 (version 2.1.0) with default parameters. SAMtools (version 0.19) was used to sort and index reads, and only reads with a unique mapping location in the genome were retained for further analysis. Htseq was used to generated count data for each transcript, and the expression of genes was normalized with fragments per kilobase of exon model per million mapped fragments (FPKM). Differential expression analysis was performed using DESeq2 (version 3.11). Genes with log_2_ (fold change) > 1 or log_2_ (fold change) < −1 and with statistical significance (*p*-value < 0.05) were deemed as DEGs.

### GO and KEGG Analysis

Goat genes were transformed into homologous human genes by biomaRt R package (version 2.40.5). GO annotation and KEGG pathway enrichment analyses of upregulated and downregulated DEGs were conducted separately using clusterProfiler R package (version 3.12.0). GO and KEGG terms with an FDR adjust *p*-value < 0.05 were deemed statistically significant.

### Gene Set Enrichment Analysis

Gene Set Enrichment Analysis (GSEA) was performed using the GSEA software and additional resources within the software. Briefly, a tab-delimited file that contains DEGs was generated and used as expression dataset. The additional resources c2.cp.kegg.v7.1.symbols.gmt and c5.all.v7.1.symbols.gmt were selected as Gene sets database. Other parameters were used as default.

### Statistical Analysis

Statistical analysis of the gene expression was conducted by the Student’s *t*-test using SPSS software (version 24.0). Data are presented as mean ± SEM, and a *p*-value < 0.05 was considered as statistically significant.

## Results

### Identification of Maternal mRNAs During Goat MZT

**FIGURE 1:**
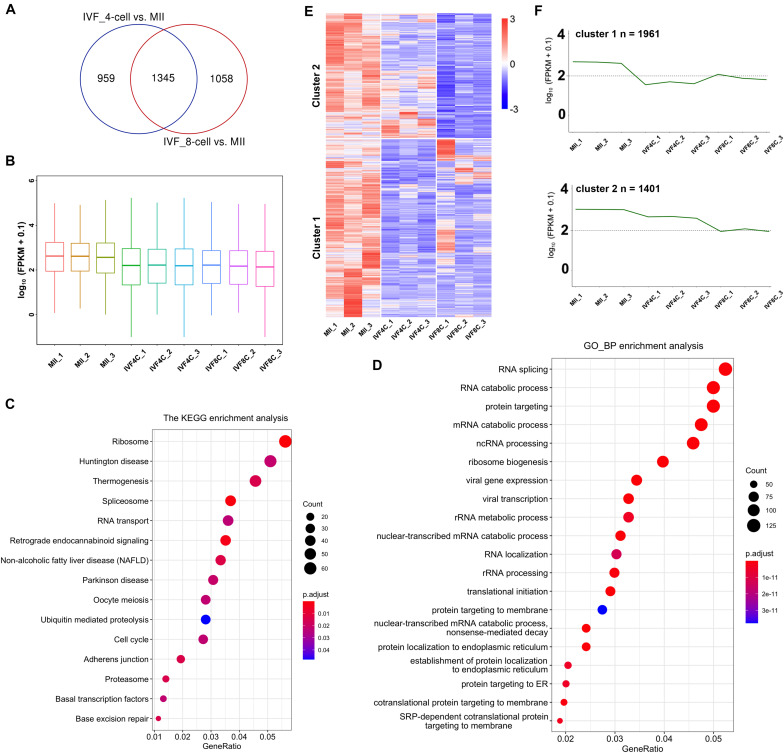
Identification of maternal mRNAs during goat MZT. **(A)** Venn plot of DEGs between the 4-cell stage embryos and the MII oocytes, and between the 8-cell stage embryos and the MII oocytes. **(B)** Boxplot of FPKM in the MII oocytes and the 4- and 8-cell stage embryos. **(C,D)** KEGG and GO enrichment analysis of the DEGs. **(E)** Heatmap of the DEGs in the MII oocytes and the 4- and 8-cell stage embryos. **(F)** Series test of cluster of the DEGs. DEGs: differentially expressed genes.

Using RNA sequencing, we identified 2304 and 2403 DEGs in the 4- and 8-cell stage embryos compared to MII oocytes, respectively. There are 1345 DEGs in both the 4- and 8-cell stage embryos (Figure 1A and Supplementary Table S3). The average FPKM in each sample in the 4- and 8-cell stage embryos was significantly decreased when compared to the samples of MII stage oocytes (Figure 1B). KEGG enrichment analysis revealed that the DEGs are enriched in ribosome, spliceosome, RNA transport, oocyte meiosis, cell cycle, basal transcription factors, and base excision repair (Figure 1C). GO enrichment analysis revealed the DEGs are enriched in RNA splicing, RNA catabolic process, and translational initiation (Figure 1D). The expression profile of the DEGs was divided into two clusters as revealed by heatmap and series test of clusters. In cluster 1, the expression of 1961 DEGs was decreased both at the 4- and 8-cell stage embryos compared to the MII stage oocytes, and slightly increased at the 8-cell stage embryos when compared to the 4-cell stage embryos (Figures 1E,F). The 1961 DEGs were termed as maternally encoded mRNA decay genes (M-decay). In cluster 2, the expression of 1401 DEGs was decreased at the 4-cell stage embryos, and further decreased at the 8-cell stage embryos (Figures 1E,F). The 1401 DEGs were termed as ZGA-dependent maternal mRNA clearance (Z-decay).

### Expression of m^6^A-Related Genes During Goat MZT

**FIGURE 2:**
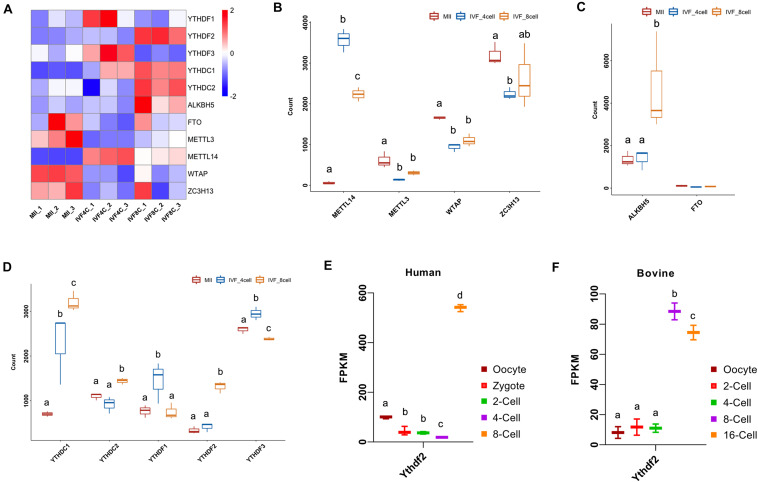
Dynamic expression of m^6^A-related genes during goat MZT. **(A)** Heatmap of the m^6^A-related genes in the MII oocytes and the 4- and 8-cell stage embryos. **(B–D)** The expression of m^6^A writers, m^6^A erasers, and m^6^A readers in the MII oocytes and 4- and 8-cell stage embryos. **(E,F)** The expression of *YTHDF2* during human and bovine MZT.

N6-methyladenosine is established by METTL3, METTl14, WTAP, and ZC3H13, and erased by ALKBH5 and FTO, and functions by direct recognition of five YTH domain-containing proteins. We next characterized the expression of m^6^A-related genes during MZT. Heatmap showed that the expression of *YTHDF1*, *YTHDF2*, *YTHDF3*, *YTHDC1*, *YTHDC2*, *ALKBH5*, *FTO*, *METTL3*, *METTL14*, *WTAP*, *ZC3H13* was dynamically changed during MZT (Figure 2A). As shown in Figure 2B, the expression of *METTL14* was increased at the 4- and 8-cell stage embryo compared with that of the MII oocytes, but was decreased at the 8-cell stage embryos when compared to the 4-cell stage embryos. The expression of *METTL3* was significantly decreased at the 4- and 8-cell stage embryos compared to the MII stage oocytes. A similar situation was found in the expression of *WTAP* during MZT. The expression of *ZC3H13* was significantly decreased at the 4-cell stage embryos but showed statistical un-change at the 8-cell stage embryos compared to the MII stage oocytes.

The expression of *ALKBH5* was not significantly changed at the 4-cell stage embryos, but strikingly increased at the 8-cell stage embryos compared to the MII oocytes (Figure 2C). The expression of *YTHDC1* was increased at the 4- and 8-cell stage embryo compared to the MII oocytes, and also increased at the 8-cell stage embryos when compared to the 4-cell stage embryos. The expression of *YTHDC2* at the 8-cell stage embryos was increased compared to the MII oocytes and the 4-cell stage embryos. The expression of *YTHDF1* was higher at the 4-cell stage embryos compared to the MII stage oocytes and the 8-cell stage embryos, a similar situation was found with the expression of *YTHDF3* (Figure 2D).

The expression of *YTHDF2* was increased at the 8-cell stage embryos compared to the MII oocytes and the 4-cell stage embryos (Figure 2D). We also investigated the expression of *YTHDF2* during embryo development in human and bovine. The expression of *YTHDF2* was gradually decreased from the oocytes to the 4-cell stage embryos, but showed a significant increase at the 8-cell embryo (ZGA stage) in humans (Figure 2E). During bovine embryo development, *YTHDF2* was consistently expressed at the oocyte, 2- and 4-cell stage embryos, and significantly upregulated at the 8/16-cell embryos (ZGA stage, Figure 2F).

### Knockdown of *YTHDF2* Impaired Goat Early Embryo Development

**FIGURE 3:**
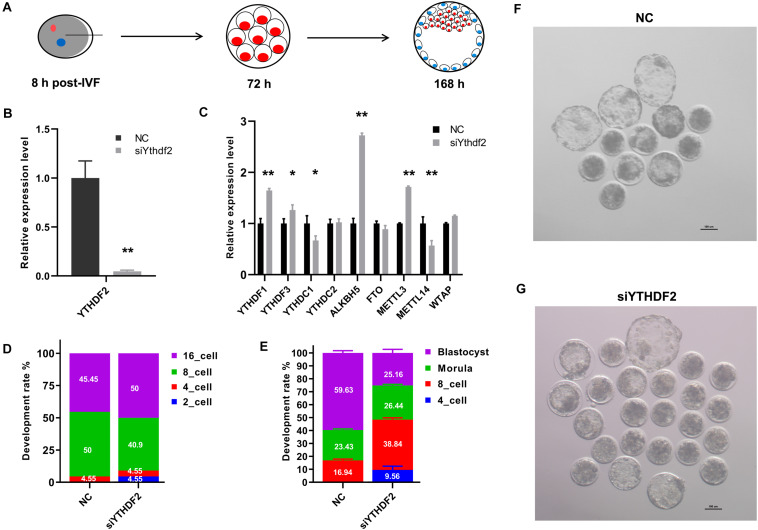
Knockdown of *YTHDF2* impaired goat early embryo development. **(A)** Schematic diagram of the YTHDF2 knockdown experiment. **(B)** The expression of YTHDF2 was significantly decreased in YTHDF2 knocked down embryos at the 8-cell stage, as revealed by qPCR. **(C)** The expression of m^6^A-related genes in YTHDF2 knocked down embryos. **(D,E)** The developmental rate at 72 h and 168 h after YTHDF2 siRNA injection. **(F,G)** Representative images of embryos in NC (*n* = 52) and YTHDF2 knocked down group (*n* = 71) at 168 h after siRNA injection.

We next investigated the role of YTHDF2 during the early embryo development in goat. Zygotes were injected with two *YTHDF2* siRNAs mixture at 8 h post-IVF. Embryo status was checked at 72 h and 168 h post-IVF (Figure 3A) and gene expression analysis was performed at the 8-cell stage embryos. The expression of *YTHDF2* was significantly decreased in *YTHDF2* knockdown embryos (Figure 3B). Moreover, the expression of *YTHDF1*, *ALKBH5*, and *METTL13* was significantly increased, while the expression of *YTHDC1* and *METTL14* was significantly decreased in *YTHDF2* knockdown embryos compared to controls (Figure 3C). Of note, 4.55% of the *YTHDF2* knockdown embryos were at the 2-cell, and the blastocyst rate was significantly decreased after *YTHDF2* knockdown (Figures 3D–G), suggesting that YTHDF2 is critical for goat early embryo development.

### Knockdown of *YTHDF2* Leads to Abnormal Gene Expression During ZGA

**FIGURE 4:**
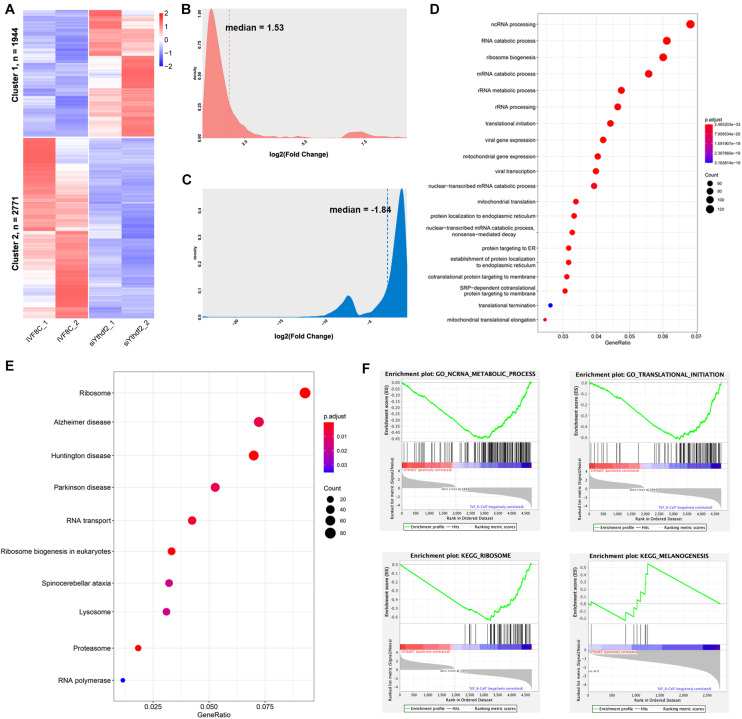
Knockdown of *YTHDF2* leads to abnormal gene expression at the 8-cell stage embryo. **(A)** Heatmap revealed 1944 genes upregulated and 2771 genes downregulated at the 8-cell stage YTHDF2 knocked down embryos. **(B,C)** Density plot of fold change of the downregulated and upregulated genes in YTHDF2 knocked down embryos at the 8-cell stage. **(D,E)** The GO and KEGG enrichment analysis of the DEGs in cluster 2 in YTHDF2 knocked down embryos. **(F)** The DEGs were enriched in non-coding RNA metabolic process, translational initiation, ribosome, and melanogenesis, as revealed by gene set enrichment analysis.

The transcription profile of YTHDF2 knockdown embryos at the 8-cell stage was investigated using RNA-seq. As shown in Figure 4A, 1944 DEGs were upregulated, while 2771 DEGs were downregulated in *YTHDF2* knockdown embryos (Supplementary Table S4). The average fold change of upregulated genes was 6.57 (Figure 4B), while that of downregulated genes was 0.35 (Figure 4C). The downregulated DEGs are enriched in ncRNA processing, RNA catabolic process, translational initiation, and mitochondrial gene expression items (Figure 4D). KEGG enrichment analysis revealed that the downregulated DEGs are enriched in ribosome, RNA transport, lysosome, proteasome, and RNA polymerase (Figure 4E). Similar results were found in the GSEA (Figure 4F). These data suggest that YTHDF2 is associated with zygotic genes expression.

### Knockdown of *YTHDF2* Disturb Maternal mRNAs Clearance

**FIGURE 5:**
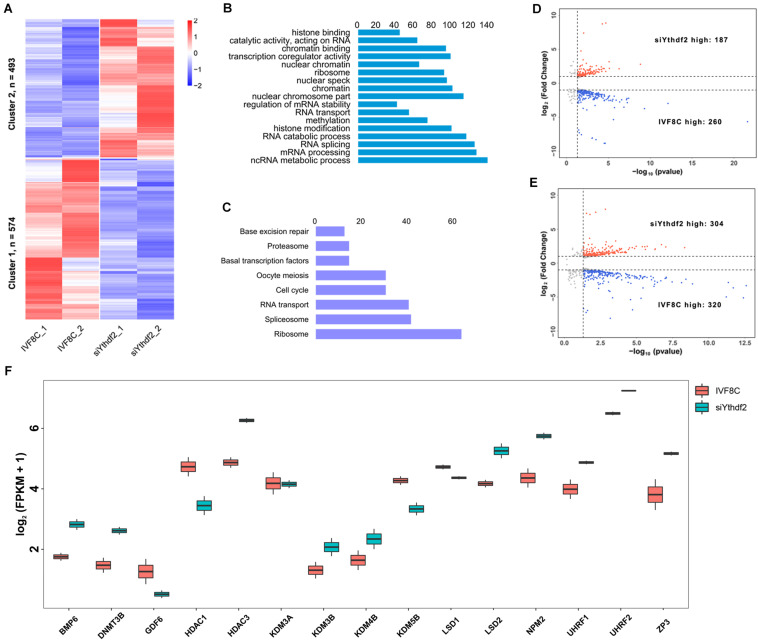
Knockdown of *YTHDF2* disturbs maternal mRNAs clearance. **(A)** Heatmap of the differentially expressed maternal mRNAs in *YTHDF2* knockdown embryos at the 8-cell stage. **(B)** The differentially expressed maternal mRNAs were enriched in RNA splicing and histone methylation items, as revealed by GO enrichment analysis. **(C)** KEGG enrichment analysis revealed that the differentially expressed maternal mRNAs were enriched in spliceosome, RNA transport, and base excision repair. **(D,E)** Volcano plot of the DEGs in ZGA-dependent maternal mRNA clearance (Z-decay) and maternally encoded mRNA decay genes (M-decay). **(F)** Boxplot of FPKM of maternal mRNAs, such as *KDM5B*, *LSD1*, and *ZP3*, in *YTHDF2* knockdown embryos at the 8-cell stage.

The expression profile of the maternal mRNAs was investigated at the 8-cell stage YTHDF2 knocked down embryos. Of the 3362 identified maternal mRNAs, 1067 genes were differentially expressed at the 8-cell stage YTHDF2 knocked down embryos. Of which, 493 genes were upregulated, while 574 genes were downregulated in *YTHDF2* knockdown embryos (Figure 5A). GO enrichment analysis revealed that the downregulated maternal mRNAs were enriched in mRNA processing, RNA splicing, regulation of mRNA stability, histone modification, histone binding, and catalytic activity, acting on RNA (Figure 5B). KEGG enrichment analysis revealed that the downregulated maternal mRNAs were enriched in ribosome, spliceosome, RNA transport, cell cycle, and oocyte meiosis (Figure 5C).

We further characterized the transcription profile of the M-decay and Z-decay maternal mRNAs. The volcano plot revealed that 260 of the Z-decay maternal mRNAs were downregulated, while 187 of which were upregulated at the 8-cell stage *YTHDF2* knockdown embryos (Figure 5D). Meanwhile, the expression of 320 M-decay maternal mRNAs was decreased, while 304 of which was increased at the 8-cell stage *YTHDF2* knockdown embryos (Figure 5E), suggesting that YTHDF2 is essential for ZGA-dependent maternal mRNA clearance. Moreover, the expression of *BMP6*, *DNMT3A*, *HDAC3*, *KDM3B*, *KDM4B*, *LSD2*, *NPM2*, *UHRF1*, *UHRF2*, and *ZP3* was significantly increased, while the expression of *GDF6*, *HDAC1*, *KDM5B*, and *LSD1* was decreased in *YTHDF2* knockdown embryos (Figure 5F), suggesting that knockdown of *YTHDF2* impaired the expression of maternal factors and crosstalk with other epigenetic modifications.

### Knockdown of *YTHDF2* Decreased the Expression of *DCP1A* and *DCP2*

**FIGURE 6:**
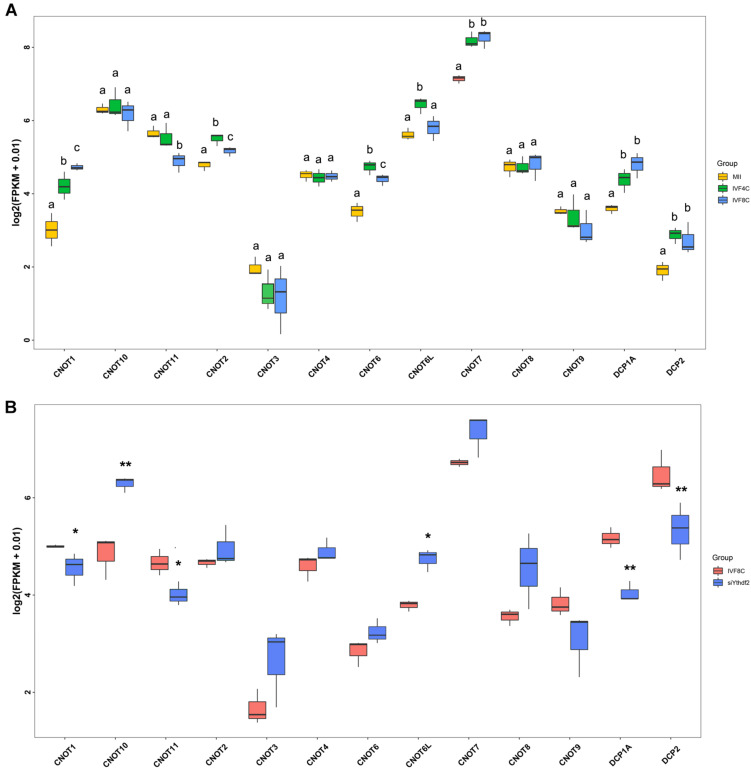
FPKM of decapping enzymes and deadenylases in *YTHDF2* knockdown embryos. **(A)** Boxplot of FPKM of decapping enzymes and deadenylases during goat MZT. **(B)** The expression of decapping enzymes (*DCP1A* and *DCP2*) and deadenylases (*CNOT1* and *CNOT11*) was significantly decreased in *YTHDF2* knockdown embryos at the 8-cell stage, as revealed by RNA-seq.

Decapping enzymes and deadenylases are responsible for maternal mRNAs degradation. To investigate the machinery that knockdown of *YTHDF2* disturbs maternal mRNAs clearance, we analyzed the expression of decapping enzymes and the deadenylases. During goat MZT, the expression of *CNOT1*, *CNOT2*, *CNOT6*, *CNOT7*, *DCP1A*, and *DCP2* was increased, while the expression of *CNOT11* was decreased at the 8-cell stage embryos compared to the MII oocytes (Figure 6A). In the 8-cell stage *YTHDF2* knockdown embryos, the expression of *CNOT1*, *CNOT11*, *DCP1A*, and *DCP2* was decreased, and the expression of *CNOT6L* and *CNOT10* was increased when compared to the controls (Figure 6B), suggesting that YTHDF2 might function through the recruitment of deadenylases and subsequent mRNA decapping during early embryo development.

## Discussion

The maternal gene products are essential for the early embryogenesis and undergo elimination during MZT in mammals (Walser and Lipshitz, 2011). Here, we identified 3362 DEGs in goat early embryo development using RNA-seq. During the MZT process, there are 3113 transcripts in mice (Wu et al., 2016), 1434 genes in humans (Yan et al., 2013), and 2169 transcripts in bovine (Bogliotti et al., 2020) whose relative abundance decreased from MII to the ZGA stage, consistent with the notion that maternal mRNA degradation is evolutionarily conserved.

Maternal mRNA clearance is dependent on the onset of ZGA in mice (Sha et al., 2019b). In the present study, we found that 1961 of the DEGs initiated degradation after fertilization and remained lower expression during MZT process, meanwhile, 1401 of the differentially expressed genes were further downregulated from the 4- to 8-cell stage embryos, which was the ZGA in goat, suggesting that degradation of these 1401 maternal mRNAs are ZGA-dependent, consistent with the results in mice (Medvedev et al., 2008; Sha et al., 2019b). Moreover, these maternal mRNAs are enriched in RNA transport, spliceosome, RNA splicing, and RNA catabolic process. A similar result was reported in the study of Jiang et al. (2014). Given that majority of maternal mRNAs were eliminated and zygotic genes onset transcription during MZT (Lee et al., 2014; Schulz and Harrison, 2019; Vastenhouw et al., 2019), it is reasonable to find that these maternal mRNAs are enriched in the pathways and GO items.

During MZT, maternal mRNAs undergo deadenylation and capping, which subsequently leads to their destructions (Garneau et al., 2007; Goldstrohm and Wickens, 2008). Consistently, we found upregulation of *CNOT1*, *CNOT2*, *CNOT6*, *CNOT7*, *DCP1A*, and *DCP2* during goat MZT. Although we could not confirm the protein expression of the deadenylases and decapping enzymes by either immunoblotting or immunocytochemistry, the data presented here indicate that the deadenylases and decapping enzymes were in the tight control of maternal mRNAs degradation in goat, and the machinery is conserved across species (Goldstrohm and Wickens, 2008; Ma et al., 2015).

N6-methyladenosine is the most abundant internal mRNA modification, which is indispensable for pluripotency maintaining in embryonic stem cell and preimplantation development, as demonstrated by the loss function of METTL3/14 (Geula et al., 2015; Xia et al., 2018; Meng et al., 2019). In the present study, we showed that knockdown of *YTHDF2* leads to an anomalous higher percentage of the 8-cell stage embryos and a lower percentage of the blastocyst in goat *in vitro*. Previously, Ivanova et al. reported that knockout of YTHDF2 increased the percentage of abnormal 2-cell embryos, which is the ZGA stage in mice (Ivanova et al., 2017), and Zhao et al. found that zebrafish embryos knocked out of YTHDF2 failed to initiate timely MZT, undergo cell cycle pause, and remain developmentally delayed throughout larval life (Zhao et al., 2017), consistent with data in our current study. Although YTHDF2 plays vital roles during MZT, the expression of YTHDF2 was relatively low before ZGA but upregulated during ZGA in bovine (Jiang et al., 2014), humans (Tang, 2010), mice (Wu et al., 2016), and our study. The inconsistency may be caused by the fact that maternal YTHDF2 might also plays pivotal roles during MZT in mammals.

YTHDF2 plays vital role in regulating transcript dosage across oocyte maturation, which is essential for generating matured oocytes that are competent to sustain early zygotic development. In the present study, we found that knockdown of YTHDF2 in goat zygotes led to abnormal expression of 4715 genes. Specifically, 2771 DEGs were downregulated and enriched in ncRNA processing, RNA catabolic process, and translational initiation, consistent with the role of YTHDF2 in transcript dosage regulation. Of note, 1067 maternal genes were aberrantly expressed in *YTHDF2* knockdown embryos, consistent with the results in mice (Ivanova et al., 2017). Moreover, we found abnormal expression of both M-decay maternal mRNAs and Z-decay maternal mRNAs. Specifically, the expression of *KDM5B* and *LSD1* was decreased. Previous studies revealed that both KDM5B and LSD1 are essential for ZGA and subsequent embryo development (Ancelin et al., 2016; Dahl et al., 2016; Glanzner et al., 2020), suggesting that YTHDF2 might control the early embryogenesis by regulating the expression of other maternal factors.

In somatic cells, YTHDF2 has been shown to function through the recruitment of deadenylases and subsequent mRNA decapping (Wang et al., 2014; Du et al., 2016). Consistent with these studies, the expression of decapping enzymes *DCP1A* and *DCP2*, and deadenylase *CNOT1* and *CNOT11* was decreased in *YTHDF2* knockdown embryos. CNOT1 is commonly involved in mRNA decay and transcriptional regulation (Paul et al., 2011), and CNOT1 knockout mice died during embryonic development (Shirai et al., 2014). CNOT11 is associated with the N-terminal region of CNOT1 (Bawankar et al., 2013), and its expression was also downregulated in our current study. We believe the downregulation of decapping enzymes and deadenylase disturb the process that YTHDF2 degrades the bound transcripts selectively during ZGA. Moreover, GO enrichment analysis revealed that the DEGs are involved in methylation, histone binding, chromatin binding, and histone methylation, it is of high tempt to hypothesis that YTHDF2 might cross-talk with histone methylation in regulation of early embryogenesis.

## Conclusion

We ascertained 3362 maternal mRNAs and inferred that maternal mRNA clearance is also ZGA-dependent in goat. We reported that YTHDF2 is important for normal development of goat embryos as it advances maternal mRNA clearance, which might through the recruitment of deadenylases and mRNA decapping enzymes. This work will be of great value for understanding the stochastic developmental events during MZT and achieving better development of goat embryos *in vitro*.

## Data Availability Statement

We acknowledged that the data presented in this study must be deposited and made publicly available in an acceptable repository, prior to publication. Frontiers cannot accept a manuscript that does not adhere to our open data policies. The accession number of the RNA-seq datasets reported in this paper is GEO: GSE156924.

## Ethics Statement

The animal study was reviewed and approved by Institutional Animal Care and Use Committees at Nanjing Agricultural University. Written informed consent was obtained from the owners for the participation of their animals in this study.

## Author Contributions

MD and FW conceptualized the experiments. MD was responsible for conducting the experiments, data analysis, and writing the manuscript. BC, YW, ZL, YC, GZ, YZ, and YF assisted with embryo production experiments, data collection, and analysis. BC and FW participated in data analysis and edited the manuscript. All authors contributed to the manuscript’s final review and editing.

## Conflict of Interest

The authors declare that the research was conducted in the absence of any commercial or financial relationships that could be construed as a potential conflict of interest.

## Supplementary Material

Supplementary Figure S1 Analysis of YTHDF2 protein homology among goat, mice, and human.

Supplementary Table S1 Sequences of siRNAs used for *YTHDF2* knockdown.

Supplementary Table S2 Details of primer sequences, expected product size, and annealing temperature (Tm,°C) of genes used for quantitative PCR.

Supplementary Table S3 FPKM of DEGs during goat MZT.

Supplementary Table S4 List of DEGs in YTHDF2 knockdown embryos at the 8-cell stage.
