# Supplementary figures and images for: YTHDF2 Regulates Maternal Transcriptome Degradation and Embryo Development in Goat
Source: Front Cell Dev Biol. 2020 Sep 29;8:580367. doi: 10.3389/fcell.2020.580367 (PMC7552740; doi:10.3389/fcell.2020.580367)

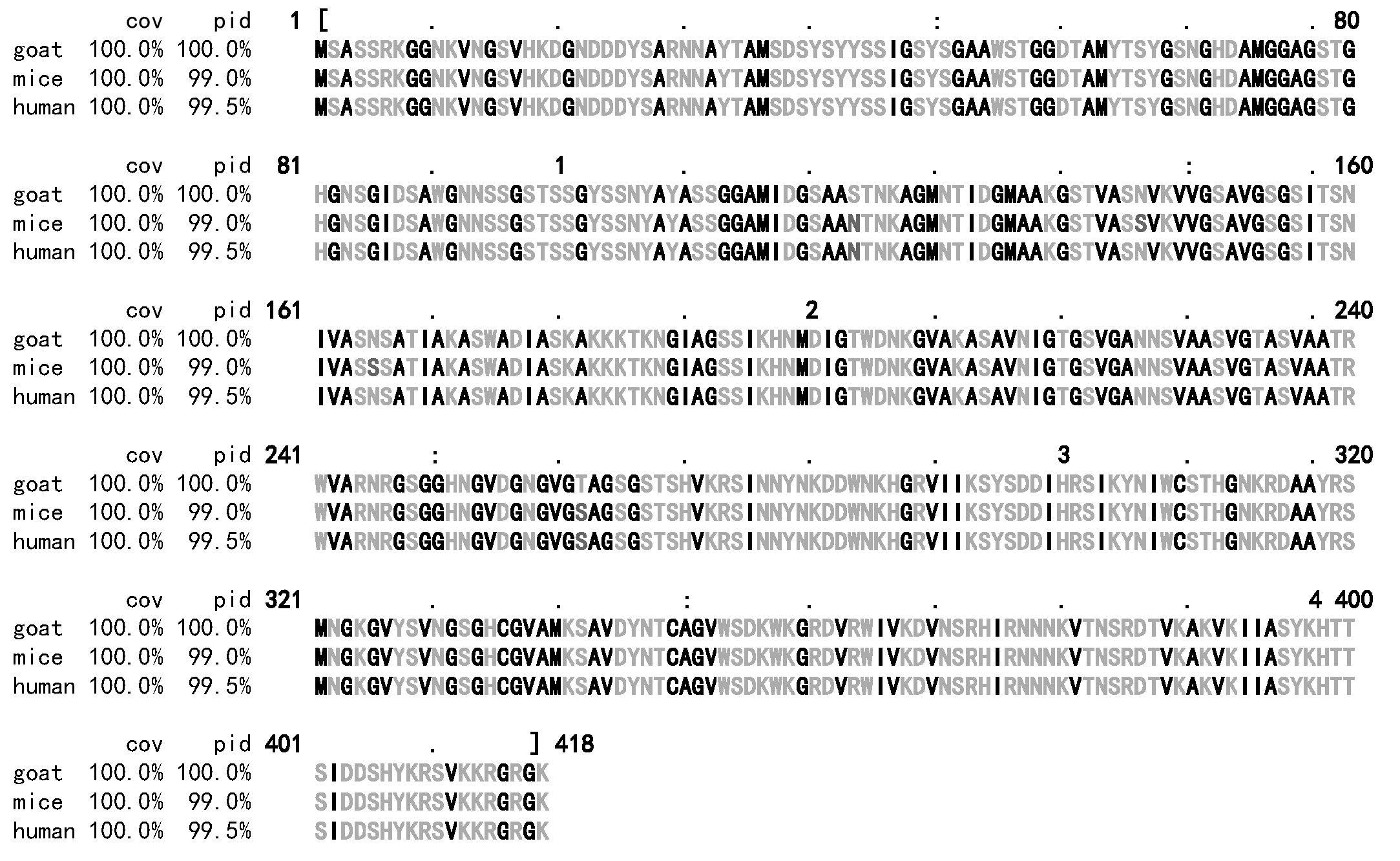

Supplement: Supplementary Figure S1 — Analysis of YTHDF2 protein homology among goat, mice, and human. [file Image_1.TIF]
