# Supplementary material for: YTHDF2 Regulates Maternal Transcriptome Degradation and Embryo Development in Goat
Source: Front Cell Dev Biol. 2020 Sep 29;8:580367. doi: 10.3389/fcell.2020.580367 (PMC7552740; doi:10.3389/fcell.2020.580367)
Supplement: Supplementary Table S1 — Sequences of siRNAs used for YTHDF2 knockdown. [file Table_1.DOCX]

**Table 1. Sequences of siRNAs used for YTHDF2 knockdown.**

| Targets | Sense sequence (5‘-3’) | Antisense sequence (5‘-3’) |
| --- | --- | --- |
| s28148 | GGGCUGAUAUUGCUAGCAATT | UUGCUAGCAAUAUCAGCCCAA |
| s28147 | GCAUGAAUACUAUAGACCATT | UGGUCUAUAGUAUUCAUGCCA |

**Table 2. Details of primer sequences, expected product size, and annealing temperature (Tm, °C) of genes used for quantitative PCR.**

| *Target gene* | Primer sequence (5‘-3’) | Product size (bp) | Accession number | Tm (°C) |
| --- | --- | --- | --- | --- |
| *YTHDC1* | F-GTCTTTAAGCCCCAACGGGA  R-CACCGTGGCCTCTACACTTA | 135 | XM_018049515 | 60 |
| *YTHDC2* | F-GTTGGTCTGAGGGATCGCAT  R-TGCCCTGCCTTTTCGTTGTA | 315 | XM_018053734 | 60 |
| *YTHDF1* | F-GGGAGGGGTTCTCGTGGATA  R-GAGGTAGGGGATTGGAGGGT | 230 | XM_018057565 | 60 |
| *YTHDF2* | F-CACAGGCATCAGTAGGGCAA  R-TTATGACCGAACCCACTGCC | 228 | XM_018057048 | 60 |
| *YTHDF3* | F-AGAGTGGAGAGCGGAAGGTC  R-TGAGAAGCCAGAAAGACGGC | 172 | XM_018058465 | 60 |
| *METTL3* | F-TGAGGCTCCTGGAAGCAAAG  R-TGCGCTGCAGTTGATTTGTC | 237 | XM_005685358 | 60 |
| *METTL14* | F-GAGAAATTGGCGCAAGGGTT  R-ACTTTCAGCTCCCAACTGCT | 149 | XM_005681276 | 59.5 |
| *WTAP* | F-CGACTAGCAACCAAGGAGCA  R-GACGCCATCAGGCCTCTATC | 233 | XM_018053431 | 60 |
| *ALKBH5* | F-AGGATTTGTGGGAGCAGAGC  R-TCGTCTCTTTTGAGGCCACC | 376 | XM_005686170 | 60 |
| *FTO* | F-GCTTTAGTTCCACCCACCGA  R-GGGGACCCCCTCTCTTCTAA | 335 | NM_001319276 | 60 |
| *Gapdh* | F-CGACTTCAACAGCGACACTCAC  R-CCCTGTTGCTGTAGCCCAATTC | 118 | NM_001034034 | 58.0 |
